# The Frequency of Malaria Is Similar among Women Receiving either Lopinavir/Ritonavir or Nevirapine-based Antiretroviral Treatment

**DOI:** 10.1371/journal.pone.0034399

**Published:** 2012-04-03

**Authors:** Tina S. Skinner-Adams, Alice S. Butterworth, Kimberly A. Porter, Ronald D'Amico, Fred Sawe, Doug Shaffer, Abraham Siika, Mina C. Hosseinipour, Elizabeth Stringer, Judith S. Currier, Tsungai Chipato, Robert Salata, Shahin Lockman, Joseph J. Eron, Steven R. Meshnick, James S. McCarthy

**Affiliations:** 1 Infectious Diseases Division, Queensland Institute of Medical Research, Brisbane, Queensland, Australia; 2 School of Medicine, University of Queensland, Brisbane, Queensland, Australia; 3 Department of Epidemiology, Gillings School of Global Public Health, University of North Carolina, Chapel Hill, United States of America; 4 Global Medical Affairs, Abbott Laboratories, New York, United States of America; 5 Kenya Medical Research Institute/Walter Reed Project, Kericho, Kenya; 6 Department of Internal Medicine, Moi University Faculty of Health Sciences, Eldoret, Kenya; 7 University of North Carolina Project, Kamuzu Central Hospital, Lilongwe, Malawi; 8 Center for Infectious Disease Research in Zambia (CIDRZ), University of Alabama at Birmingham, Birmingham, United States of America; 9 UCLA CARE Center, University of California Los Angeles, Los Angeles, California, United States of America; 10 University of Zimbabwe, Harare, Zimbabwe; 11 Division of Infectious Diseases Department of Medicine, Case Western Reserve University, Cleveland, Ohio, United States of America; 12 Division of Infectious Diseases, Brigham and Women's Hospital, Boston, Massachusetts, United States of America; 13 Division of Infectious Diseases, School of Medicine, University of North Carolina, Chapel Hill, North Carolina, United States of America; National AIDS Research Institute, India

## Abstract

HIV protease inhibitors (PIs) show antimalarial activity *in vitro* and in animals. Whether this translates into a clinical benefit in HIV-infected patients residing in malaria-endemic regions is unknown. We studied the incidence of malaria, as defined by blood smear positivity or a positive *Plasmodium falciparum* histidine-rich protein 2 antigen test, among 444 HIV-infected women initiating antiretroviral treatment (ART) in the OCTANE trial (A5208; ClinicalTrials.gov: NCT00089505). Participants were randomized to treatment with PI-containing vs. PI-sparing ART, and were followed prospectively for ≥48 weeks; 73% also received cotrimoxazole prophylaxis. PI-containing treatment was not associated with protection against malaria in this study population.

## Introduction

The majority of HIV-infected individuals live in malaria-endemic regions, and available evidence suggests that the interaction between HIV and malaria infection is complex, synergistic and bidirectional. Reported effects include increased HIV replication resulting from immune activation [1], increased parasitaemia (likely due to attenuated malaria-specific immunity) [2], and complex pharmacokinetic interactions between antiretroviral and antimalarial drugs [3], [4]. Taken together these interactions likely result in worse clinical outcomes for co-infected patients [5], [6].

HIV protease inhibitors (PIs) have moderate *in vitro* activity against the two most important malaria parasites, *Plasmodium falciparum* [7] and *P. vivax* [8], as well as *in vivo* activity against rodent malaria parasites [9]. The antimalarial action of these agents is not fully understood but is likely related to their inhibition of malaria parasite aspartic proteases or plasmepsins [10]. In addition to their antiretroviral activity, PIs have been reported to affect CD36-mediated cytoadherence of *Plasmodium falciparum*-infected erythrocytes [11]. These data suggest that PIs may afford antimalarial protection in HIV-infected people residing in malaria endemic regions. However, whether this activity translates into a clinically relevant effect in endemic settings remains to be determined.

To explore the effect of PI-based ART on HIV-infected people residing in malaria-endemic regions of sub-Saharan Africa we undertook a retrospective study examining the incidence of malaria in a group of women enrolled in AACTG 5208 [12]. In this analysis, records of clinical diagnoses of malaria were not included as these may lack sensitivity by missing subclinical infection (malaria parasitaemia without malaria symptoms), and specificity, whereby non-specific febrile illnesses commonly attributable to malaria may be caused by other conditions [13]. Instead we compared the incidence of malaria in women randomized to start ART with either a PI-containing or a PI-sparing regimen in the OCTANE (A5208), with malaria diagnoses defined by either slide-confirmed diagnoses or by confirming the presence of circulating parasite antigen in stored blood samples.

Detection of *P. falciparum* histidine-rich protein two (HRP2) by antigen-capture immunoassay forms the basis of most rapid diagnostic tests for falciparum malaria. This water-soluble protein is released into the circulation of individuals infected with *P. falciparum*, and has been shown to persist in the circulation after clinical symptoms of malaria have resolved and parasites have been cleared from the circulation. It has been reported to remain in the blood for 7 days following treatment in 62–99% of patients, for 14 days in 35–98% of patients, and for 28 days in 27–92% [14], [15].

## Methods

The OCTANE study (AACTG 5208) consisted of two concurrent randomized trials; detailed methods and primary results have been published [12]. In Trial 1 (N = 243), women who had previously received single-dose nevirapine (NVP) were studied, whereas Trial 2 participants were women with no history of NVP exposure (N = 502). In both trials, women were randomized 1∶1 (stratified by screening CD4+ cell count: <50 cells/µL or ≥50 cells/µL) to receive either ritonavir-boosted lopinavir (LPV/r)-based ART or nevirapine (NVP) -based ART. All participants also received tenofovir disoproxil fumarate and emtricitabine. Participants visited study sites at regular intervals (0, 4, 8, 12, 16, and 24 weeks; then every 12 weeks) for health assessments that included collection and storage of venous blood, and were followed for at least 48 weeks. The protocol and consents were approved by IRBs at each site, and each participant provided written informed consent.

The current study includes data obtained from participants enrolled at the six OCTANE study sites where malaria transmission has been reported to occur (Eldoret, Kenya, Kericho, Kenya; Lilongwe, Malawi; Kampala, Uganda; Lusaka, Zambia; and Harare, Zimbabwe). Study subjects were considered to have malaria if they had a positive blood smear, a positive malaria rapid diagnostic test (RDT), or if malaria antigen (*P. falciparum* Histidine-rich protein 2 [HRP2]) was detected in the plasma samples that were collected at study visits. All available samples from each participant at the relevant sites were tested for HRP2 antigenemia using a commercially available ELISA kit (Standard Diagnostics, SD malaria Antigen Pf ELISA). Each plasma sample was tested in duplicate as stated in the manufacturer's instructions with positive and negative controls on every plate. Evaluation of this kit before the study indicated that it was more sensitive than other commercially available plate-based HRP2 ELISA kits and was between 30 and 120 fold more sensitive than malaria rapid diagnostic kits (data not shown).

As malaria was not detected in two of the study sites and some participants changed ART regimen and/or received cotrimoxazole, a drug with known prophylactic activity against malaria in HIV-infected populations [16], in a non-uniform fashion during the study, data were analyzed on a cross-sectional basis and stratified by treatment arm and cotrimoxazole use at the time of sample collection. To avoid counting two sequential samples from the same infected patient as two episodes, positive tests from a single patient were counted as single episodes unless they were separated by a negative sample. Data were tested for statistical significance using a chi-squared test (http://www.openepi.com). Multivariate analyses were not performed given the small numbers of samples with a confirmed diagnosis of malaria.

## Results

HRP2 antigenemia was assessed by examining available plasma samples totaling 3,516 from 444 women. All available samples where a diagnosis of malaria had been made by blood smear, on-site rapid diagnostic test or HRP2 ELISA where also tested for malaria in our laboratory using immunochromatographic rapid diagnostic tests (RDTs; First Response; Premier Medical Corporation Limited and First Sign ParaView; Unimed). An additional 113 samples from these 444 women were tested for malaria during the original study (on site blood smear or RDT). As blood smear slides were not retained these diagnoses could not be revalidated. Therefore, laboratory data for malaria were available from a total of 3629 samples from 444 subjects (1985 samples from women in the LPV/r arm and 1644 from the NVP arm, Table 1).

**Table 1:** Episodes of *P. falciparum* malaria (positive on-site smear or RDT, or positive *Pf* Histidine-rich Protein-2 (*Pf*HRP2) antigen test) in the 6 malaria-endemic study sites.

Harare(Zimbabwe)690 samples(115 subjects)		Eldoret(Kenya)438 samples(64 subjects)		Kericho(Kenya)563 samples(73 subjects)		Kampala(Uganda)524samples(60 subjects)		Lilongwe(Malawi)641 samples(68 subjects)		Lusaka(Zambia)773 samples(64 subjects)	
PI-ART*		PI-ART		PI-ART		PI-ART		PI-ART		PI-ART	
Yes370	No320	Yes243	No195	Yes317	No246	Yes270	No254	Yes340	No301	Yes445	No328

Cotrimoxazole				Cotrimoxazole				Cotrimoxazole				Cotrimoxazole				Cotrimoxazole				Cotrimoxazole			
Yes113	No257	Yes100	No220	Yes167	No76	Yes156	No39	Yes268	No49	Yes218	No28	Yes213	No57	Yes167	No87	Yes272	No68	Yes268	No33	Yes367	No78	Yes254	No74

Malaria				Malaria				Malaria				Malaria				Malaria				Malaria			
0	0	0	0	0	0	0	0	4	2	1	0	0	0	2	2	16	1	6	0	38	13	13	6
0%	0%	0%	0%	0%	0%	0%	0%	1.5	4.1	0.5%	0%	0%	0%	1.2%	2.3%	5.9%	1.5%	2.2%	0%	10.4%	16.7%	7.7%	8.1%

*P. falciparum* infection was confirmed in 104 (2.9%) of the 3629 samples tested (Table 1), 34 by on-site blood smear or RDT, and 71 by HRP2 antigenemia. Only ten of the positive on-site samples were available for retrospective testing for HRP2 antigen, one of which tested positive. All samples that tested positive with RDTs also tested positive for HRP2 by ELISA. No confirmed cases of malaria were identified at the Harare (Zimbabwe) or Eldoret (Kenya) sites (table 1). Subsequent analysis was therefore restricted to the four sites where confirmed diagnoses were made (Table 2). A total of 2,388 samples were tested from 265 women enrolled at these four sites, among whom 53 (20%) had one or more episode of confirmed malaria over the course of the study. The proportion of samples positive for malaria was low, with 104 positive tests (4.6% of samples). The distribution of these positive tests varied widely across the four study sites (Lilongwe, 23/641 (3.6%); Kampala 4/524 (0.8%); Kericho 7/563 (1.2%); Lusaka 70/773 (9.0%). A greater proportion of samples taken from subjects receiving LPV/r-based ART were positive for malaria compared to samples taken from subjects receiving vs. NVP-based ART although the difference was not statistically significant (2.8% compared to 1.8%, respectively, p = 0.13; Table 2A).

**Table 2:** Malaria diagnoses made at the four sites where malaria was detected by blood smear (on-site testing), RDT (on-site testing) or *Pf* Histidine-rich Protein-2 (*Pf*HRP2) antigenemia.

A		B	
# (%) of patients with at least one episode of malaria(N = 2268 samples from 265 patients)		Episodes of malaria(N = 2462 samples from 265 patients)	
PI-ART		PI-ART	
Yes1221	No1047	Yes1342	No1120

Cotrimoxazole				Cotrimoxazole			
Yes1007	No214	Yes843	No204	Yes1095	No247	Yes899	No221

Malaria				Malaria			
#27(2.7%)	#7(3.3%)	#14(1.7%)	#5(2.4%)	#33(3.0%)	#11(4.4%)	#15(1.7%)	#6(2.7%)

A) ACTG 5208 study participant samples were censored to include only one malaria diagnosis per patient; B) ACTG 5208 study participant samples to include multiple episodes (only if separated by at least one malaria negative sample).

Consistent with standard management guidelines for patients infected with HIV, the majority (81%) of study subjects were taking daily cotrimoxazole prophylaxis at the time of blood sampling (Table 1). While the incidence of malaria (allowing only one episode of malaria per subject; 2.9% versus 2.2%; Table 2A) and the number of episodes of malaria (allowing for multiple episodes per patient only if separated by a negative sample; 3.6% versus 2.4%; Table 2B) were slightly higher among subjects not taking cotrimoxazole prophylaxis at the time of blood sampling, this was not significant (P = 0.42 and P = 0.14 respectively). When the combined effect of cotrimoxazole therapy and the antiretroviral regimen was examined, the incidence of malaria was lowest among samples drawn from subjects taking cotrimoxazole and NVP-based ART (1.7%; Table 2A) compared to those drawn from patients taking cotrimoxazole and LPV/r-based ART (2.7%). Subjects not taking cotrimoxazole also had a higher incidence of malaria when taking LPV/r-based ART (3.3%) than those subjects taking NVP-based ART (2.4%). None of these differences were significant (P = 0.39).

## Discussion

In this study, we observed a low incidence of malaria as defined by positive malaria smears, RDTs, and antigenemia, and did not find that PI-based ART was associated with a lower incidence of malaria. Fifty three (20%) of the women in the study had at least one episode of laboratory-confirmed malaria over the study interval, 34 (64%) of these women were receiving PI-based ART at the time of malaria diagnosis (Table 2A).

Although the results indicate that PI therapy did not exert a protective effect against malaria in this population, a number of circumstances that may have masked a protective effect should be considered. In the first instance, it is possible that the low numbers of malaria diagnosis made in our cohort underpowered the study and prevented the identification of a protective effect. While this study was predicted to have sufficient power to answer our question when it was first designed, there was a significant reduction in the prevalence of malaria in many parts of Africa in the interval between design and study execution [16]. An additional analysis where the incidence of malaria in this study population was defined on clinical grounds rather than using a more rigorous definition requiring a laboratory confirmed diagnosis was in agreement with results reported here (manuscript submitted).

The high rate of cotrimoxazole prophylaxis (80%) observed in our study population, an intervention with a protective effect against malaria in HIV-infected populations, may have also impacted our results by reducing the episodes of malaria. While pharmacokinetic interactions are also possible when drugs are co-administered, and are a particular concern with ritonavir, a drug well known to affect the pharmacokinetics of many drugs including antimalarials, an interaction of this drug with cotrimoxazole seems unlikely to be an explanation for any lack or antimalarial effect observed among subjects in the current study. Both groups receiving cotrimoxazole, regardless of their ART treatment regimen had reduced episodes of malaria (Table 2).

A pharmacodynamic interaction may be associated with the inability of PI-based ART to protect the current cohort of women from malaria infection. While the primary mode of action of cotrimoxazole is understood, the antimalarial mode of action of the PIs is still not known. A better understanding of the basis of the antimalarial effect of PIs may enable prediction of clinically significant pharmacodynamic interactions. Both cotrimoxazole and individual PIs may also have off-target effects that alter the activity of the other drug or additional drugs that may be used to treat HIV-infected patients with opportunistic pathogens. These interactions must be understood to ensure the use of the most effective drug treatment regimens. The ability of the PIs to modulate aspects of the immune system, decrease the expression of CD36 and interfere with the phagocytosis of parasitized erythrocytes [11], may also be associated with the inability of LPV/r to provide antimalarial protection in the current study.

Indirect antimalarial benefits, that were not examined in this study, including reduced cytoadherence [11] and decreased gametocyte carriage [17] that may also be derived from PI-based ART also need to be considered. Studies in children and pregnant women, who are at greater risk of clinical malaria, also need to be performed. Additionally, as laboratory studies suggest that the PIs interact synergistically with selected antimalarial agents such as chloroquine and mefloquine [18], the use of these combinations warrants further investigation. These combinations may be particularly useful against multi-drug resistant (MDR) parasites given the ability of PIs to inhibit MDR pumps [19]. They may also result in the boosting of drug levels due to cytochrome P450 interactions/competition [20].
